# Supplementary material for: Are carbon emissions trading and green financial instruments synergistic? -Comprehensive quantitative research based on content analysis
Source: PLoS One. 2024 Mar 7;19(3):e0298601. doi: 10.1371/journal.pone.0298601 (PMC10919601; doi:10.1371/journal.pone.0298601)
Supplement: S1 Data — (ZIP) [file pone.0298601.s001.zip › Data/地级市Z_F工作报告.zip!/╣π╢1⁄2/│▒╓▌╩╨/└┤╫╘╣1⁄2╓┌║┼í╛┬φ┐╦╩2╛▌═°í┐_2018.docx]

各位代表：

现在，我代表市人民政府向大会作工作报告，请予审议，并请市政协委员和其他列席人员提出意见。

2017年工作回顾

过去一年，是我市凝心聚力抓建设、砥砺奋进促发展、坚定不移惠民生的一年。在省委、省政府和市委的正确领导下，在市人大及其常委会和市政协的监督支持下，市政府坚持以习近平新时代中国特色社会主义思想为指引，深入落实党的十八大、十八届中央历次全会精神，认真贯彻落实党的十九大精神、习近平总书记对广东工作的重要指示批示精神以及省第十二次党代会、市第十四次党代会精神，以加快振兴发展为主题，以供给侧结构性改革为主线，依托“三大抓手”，聚焦实体经济，狠抓重点项目，锐意改革攻坚，深推惠民共享，有力促进经济社会平稳健康发展。据初步测算，全市实现生产总值1075亿元，突破千亿大关，比上年增长7%；人均生产总值4万元左右，增长6.8%；一般公共预算收入44.6亿元，增长1.9%；各项税收总收入95.9亿元，增长6.1%；规模以上工业增加值405亿元，增长6.9%；固定资产投资500亿元，增长10%；外贸进出口总额211.5亿元，增长5.8%；社会消费品零售总额545亿元，增长10%；城乡居民人均可支配收入1.9万元，增长7%；居民消费价格指数上涨1.4%。

一、项目建设成效显著

全力推进项目建设“百团大战”，全市96个市重点项目完成年度投资181.3亿元，占计划的110%。一是交通扩网取得新飞跃。成功破解外环西路等多个征拆难题，创造了项目征交地的“大潮速度”，全年完成交通项目征地9169亩、拆迁28.3万平方米。22个新建、改扩建道路桥梁项目完工通车，创下我市交通建设历史新高。广梅汕铁路增建二线及厦深联络线、梅汕客专等项目加快推进，厦深铁路饶平站实现始发运营。高速公路建设成效空前，甬莞高速潮州段全线通车，高速公路通车里程实现翻番，达到130公里。潮州大桥、甬莞高速韩江特大桥、凤泉湖大桥三桥同日合龙，并与如意大桥先后实现通车。绿榕北路等新改建道路顺利完工，全市交通路网日臻完善。二是平台建设取得新成效。坚持规划先行，引领推动城市多核化发展，城市总体规划和凤泉湖高新区等重点区域控规编制完成。潮州新区起步区27个重点项目完成年度投资43.6亿元，东方国际茶都等项目投入使用，中国工艺美术之都博览园、总部基地等项目启动实施。凤泉湖高新区二期万亩征地进展顺利，与铁铺镇“园镇一体化”有效落实，中山大道全线贯通,公租房等配套逐步完善，园区腹地成10倍扩展。高铁新城完成核心区征地工作，园区规划编制和基础设施建设全面启动。闽粤经济合作区规划、征地、建设工作扎实推进，潮州港亚太通用码头一期工程通过验收。三是民生项目取得新突破。“六个十”项目建设如火如荼，一批公园、肉菜市场、“黑底化”道路项目相继竣工，我市成为广东省唯一一个全国“厕所革命优秀城市”。电力保障全面加强，年度电网投资11.8亿元，增长66.4%。172宗村村通自来水工程完成建设。征地留用地历史遗留问题等16项短板事项成功解决，村卫生站公建规范化建设进度居全省首位。四是水利工程取得新进展。全市水利建设投入20亿元，韩东新城防洪、北堤、南堤首段等综合整治工程全面动工，粤东灌区续建配套与节水改造工程有序推进。

二、产业质效持续优化

深入推进供给侧结构性改革，全面实施创新驱动发展战略，实体经济不断壮大，产业质量和效益明显提升。一是产业实力更强。568家企业实现“个转企”，规上企业增至890家，上市和挂牌企业分别增至9家和47家，产值亿元以上工业企业达到456家，增加116家，认定“四梁八柱”企业44家。二是创新水平更高。先进陶瓷材料创新研究中心成功建立，新认定省级工程中心、技术中心12家，年产值5亿元以上工业企业实现研发机构全覆盖，省级孵化器取得零的突破，广东健诚高科玻璃陶瓷项目获省科学技术奖一等奖。高新技术企业78家，增长47%，三环集团入选广东省高新技术企业百强榜。预计规上高技术制造业增加值28.9亿元，增长13.5%。制定实施“人才新政23条”，顺利建成全省首家“人才咖啡”，成立潮州市高层次人才联谊会，新引进博士、硕士199人。三是发展动能更足。招商引资深推猛进，华南最大的装配式建筑产业基地、益海嘉里粮油基地、中天丙烷脱氢、华瀛等一批百亿级产业项目加快推进，国电海山风能等21个项目建成投产。湘桥区意溪镇、饶平县钱东潮商文化小镇分别入选国家和省特色小镇创建工作示范点。联饶镇被认定为省级专业镇，总量增至21个。四是产业结构更优。预计三次产业结构调整优化为6.9:50.8:42.3。农业现代化建设步伐加快，粮食生产保持稳定，特色农业加快发展，凤凰单丛茶获得“中国优秀茶叶区域公用品牌”，凤凰镇获评“全国十大魅力茶乡”，饶平县入选全国首批农业对外开放合作试验区。旅游产业发展迅猛，全市接待海内外游客1535万人次，实现旅游收入235亿元，分别增长30.2%和29.5%。我市和湘桥区成为广东省全域旅游示范区创建单位，“品味潮州·寻梦古城”百家旅行社签约活动顺利举办，笔架山潮州窑考古遗址公园成为全省首个通过国家级考古遗址公园立项项目，饶平县青岚地质公园成为粤东首家国家级地质公园。

三、发展环境大幅改善

深入推进综合治理，切实破解制约发展的环境障碍。一是城乡治理有力推进。“治六乱”行动取得突出成效，投入治理资金3.9亿元，清理路面3.4万公里、河岸6850公里、各类垃圾约83万吨，“两违”“两清”专项整治完成清拆近110万平方米，清理禁养区规模化和专业户畜禽养殖场（户）322个。西湖夜景提升、人民广场音乐喷泉改造等69个项目全面完工，中山路工艺街景观提升、一江两岸夜景提升等15个项目顺利实施，城乡环境和市容市貌大幅优化。二是经济治理有序开展。狠抓经济户口清理，在全省率先建立工业企业基本信息库，完成企业录入审核8465家。实施“1+N”产业扶持措施，落实扶持资金3.4亿元，减免各项税费9.7亿元。推进投融资体制改革，开设“金融超市”，聚集“潮民投”“闽粤民投”和中潮产业、宜华股权投资基金等民营资本超过30亿元，促成各类融资96.8亿元。存贷比为31.6%，提高4.6个百分点。普惠金融“村村通”实现全覆盖。三是生态治理有效落实。坚决打好大气、水、土壤污染防治攻坚战，全年空气优良天数比例达到95.6%，空气质量改善幅度居全省首位，韩江获评全国十大“最美家乡河”。中央环保督察反馈意见整改有效落实，市第二污水处理厂完成一期厂区主体工程建设，潮安区生活垃圾焚烧发电厂提前建成运营。生态建设成效明显，新增60个市级生态示范村，新建省级森林小镇3个，完成造林1.7万亩、森林碳汇抚育5.4万亩。

四、改革开放纵深推进

全面深化改革、扩大开放、加强合作，不断增强经济发展的活力和动力。一是改革措施进一步落实。推进“多证合一、一照一码”等商事制度改革，开办企业便利度跃居粤东西北12市首位，新登记市场主体增长30.9%，各类市场主体突破十万户。在全省率先完成国地税联合办税服务厅共建，实现“一厅对外”“一窗办税”。二是商务贸易进一步拓展。电子商务加快发展，顺利完成饶平县电子商务进农村综合示范县建设。以建设“新外贸示范市”为抓手，打造跨境电商综合服务平台，助推进出口贸易，外贸进口与出口实现双增长。新增外商投资项目38个，合同利用外资倍增。成功举办国际婚纱礼服周、“中陶奖”、陶瓷精品展、中国刺绣艺术展等国际性、全国性活动，潮州品牌效应进一步彰显。三是区域合作进一步深化。与中山市的新一轮对口帮扶合作成效突出，“中潮慈善爱心基金”成功设立并筹款1026万元。汕潮揭同城化继续推进，《汕潮揭临港空铁经济合作区发展总体规划（2016-2030）》出台实施。

五、社会事业全面进步

扎实推进普惠共享，大力拓展公共服务的广度和深度，切实保障和改善民生。民生领域投入资金112.7亿元，占公共预算支出78.9%。一是基本保障落实到位。推进就业创业工作，新增城镇就业1.87万人，城镇登记失业率控制在2.58%。加强社保扩面征缴工作，养老、医疗、失业、工伤、生育等保险覆盖率进一步提升，社保体系更加健全。二是扶贫济困精准有效。在全省率先出台因病致贫、因病返贫医疗救助实施办法，完成精准扶贫年度脱贫任务，45个省定贫困村已落实建设资金3.9亿元。优抚对象、低保、五保、残疾人等补助标准提高，大病保险为3.3万参保人次减少费用超1亿元。三是公共服务全面深化。医疗服务水平持续提升，基层医疗机构家庭医生签约服务实现全覆盖，市中心医院新院动工建设，市妇幼保健院扩建工程加快推进，饶平县第二人民医院、饶平县急救医疗（指挥）中心和潮安区人民医院改扩建工程启动建设。教育现代化建设步伐加快，市特殊教育学校投入使用，潮州卫生健康职业学院和潮汕高等研究院建设加快推进。文化事业蓬勃发展，建成市图书馆、市档案馆及314个基层综合性文化服务中心，新增省级非遗传承基地6个、省级非遗传承人9名。第十六届市运会顺利举办。四是平安建设全面升级。建成潮州智能交通指挥中心，完成平安潮州视频监控系统二期工程建设，社会治安立体化防控体系进一步完善，总警情、刑事警情、治安警情和两抢一盗警情实现“四下降”，粤东最平安城市创建初具成效，群众满意度大幅提升。试点基层治理“四化”建设，信访综治、应急管理等工作更加扎实，社会大局和谐稳定。新出台《潮州市重大生产安全事故隐患治理挂牌督办实施细则》等6个规范性文件，进一步压实安全生产责任、健全安全监管机制，“城中村”消防整治、防范地质灾害等专项工作扎实开展，连续14年保持无重特大安全事故发生。食品药品安全保障更加有力，市食品检测中心建成交付使用。与此同时，人事、外事侨务、港澳、审计、统计、贸促、物价、粮食、打私、打假、人防、气象、防震、地方志、档案、对台、拥军优抚、民兵预备役、老龄、民族宗教、妇女儿童等工作取得新成绩。

六、政府效能全面提升

推进依法行政，狠抓体制改革，切实提高政府服务水平和效能。一是民主法治不断深化。自觉接受人大、政协监督，34件人大代表建议、92件政协委员提案全部按时办结。严格依法履职，重视和加强法制工作，切实提高行政工作规范化、科学化水平。二是行政审批不断简化。取消行政许可事项32项，清理规范行政审批中介服务事项28项，在粤东地区率先开放施工图审查市场。推行“三个三”工作法，实行容缺审批、并联审批和“绿色通道”等快速审批机制，行政审批提速率达45.8%。完善网上办事大厅建设，网上全流程办理率84.7%。三是行政体制不断优化。有序推进城乡管理执法体制改革，理顺市级文物管理体制，完成市级农业综合行政执法体制改革。四是廉政建设不断强化。加强政务公开，推进零基预算改革和预决算信息公开，落实行政处罚和行政许可“双公示”。规范“三公”经费管理，党政机关单位会议费及“三公”经费财政拨款支出下降9.1%。强化审计监督和行政监察，全面落实党风廉政建设责任制，反腐倡廉工作成效明显。

各位代表，过去一年所取得的成绩,是省委、省政府和市委坚强领导的结果,是市人大、市政协监督支持的结果，也是全市人民戮力同心、开拓拼搏，驻潮部队、武警官兵、上级驻潮各单位和海内外各界人士倾力相助的结果。在此，我代表市人民政府表示衷心感谢并致以崇高敬意！

在看到成绩的同时，我们也清醒地认识到，我市经济社会发展仍面临诸多困难和问题，发展不平衡不充分问题仍较突出，主要有：经济总量不大，产业结构不优，规模经济不强，发展质量和效益相对较低；配套设施不完善，城市管理水平不高，环境治理任重而道远，综合竞争力亟待提升；公共服务供给能力不足，与人民群众日益增长的美好生活需要还有一定差距，普惠民生的力度有待进一步增强；行政体制机制还不够完善，制约发展的习惯性、制度性问题依然存在，改革攻坚还需深入推进，政府法治意识和作风建设仍需加强。对此，我们一定高度重视，采取强有力措施加以解决。

2018年工作安排

2018年是贯彻落实党的十九大精神的开局之年，是改革开放40周年，是决胜全面建成小康社会、实施“十三五”规划承上启下的关键一年。当前，全球经济温和复苏，外需状况有所改善，但逆全球化、地缘政治等不确定性、不稳定性因素依然存在。随着党的十九大一揽子重大战略决策的实施，以及供给侧结构性改革不断释放强大动力，国内经济稳中向好的态势更加稳固，省委关于把东西两翼沿海地区打造成新增长极的战略部署，为潮州发展带来了新的更大机遇。经过不懈努力，我市发展稳的基础更加牢固、进的潜能更加强劲，干部群众干事创业的“精气神”全面提振，加快振兴发展的“势”和“能”更加充分。我们必须立足新起点，融入新时代，抢抓新机遇，凝心聚力开创发展新局面。

今年政府工作的总体要求是：高举习近平新时代中国特色社会主义思想伟大旗帜，全面贯彻党的十九大精神和中央经济工作会议、农村工作会议精神以及省委十二届二次、三次全会精神，围绕市第十四次党代会确定的总体思路和市委十四届四次全会的部署要求，坚持稳中求进总基调，坚持新发展理念，按照高质量发展的要求，统筹推进稳增长、促改革、调结构、惠民生各项工作，打好防范化解重大风险、精准脱贫、污染防治三大攻坚战，着力优化政治生态、经济生态、自然生态，促进经济社会持续健康发展，加快建设享誉中外、独具品质、宜业宜居宜游的潮文化精品城市和世界潮人精神家园。

综合各方面因素，今年我市经济社会发展的主要预期目标是：生产总值增长7%左右，一般公共预算收入增长6%，固定资产投资增长12%，外贸进出口总额增长3%，全社会消费品零售总额增长10%，居民消费价格指数涨幅控制在3%以内，居民收入增长与经济增长基本同步，城镇登记失业率控制在3.5%以内，大气、水环境质量改善完成国家、省下达目标任务，供给侧结构性改革取得实质性进展，金融财政风险有序高效防控。

为实现上述目标，我们将全力以赴抓好六方面工作。

一、聚焦创新驱动，振兴实体经济

以建设现代化经济体系为目标，以“高质量发展”为导向，把着力点放在实体经济上，深入实施创新驱动发展战略，全力增强经济质量优势。

做强创新经济。以《中国制造2025》为引领，深入落实“科技创新18条”，启动技术改造导向计划，推进工业企业大数据服务平台建设，引导企业对接互联网、人工智能，力促制造业提升数字化、智能化水平。瞄准全球陶瓷产业制高点，加快推进三环先进材料生产基地项目，抓好“工业4.0”工厂、数字化车间建设，打造国际领先的高端电子材料基地。以先进陶瓷材料创新研究中心为平台，加强与国内外研发机构产学研联动，推动科技成果加快转化。以广东陶瓷创意联盟为载体，探索构建国际陶瓷联盟，开展陶瓷价格指数编制，提升陶瓷产业的高端化、国际化水平。建立以企业为主体、市场为导向、产学研深度融合的技术创新体系，完善“市县镇企”四级创新联盟，推进中山-潮州产业创新中心建设。创建更多博士后科研平台、院士工作站，新建一批孵化器、众创空间，规模以上企业研发机构覆盖率达到30%以上。加快创新驱动先行区和国家级高新区创建步伐，加强高新技术企业培育，力争高企数量达到130家。推进知识产权保护，启动运营中国潮州（餐具炊具）知识产权快速维权中心。激发和保护企业家精神，建设知识型、技能型、创新型劳动者队伍。推动“人才新政23条”落地见效，加强与清华大学等知名高校和省直单位合作，吸引更多专业型干部到我市挂职。建立高层次人才信息库、急需紧缺人才数据库和人才储备中心，完善定向招录专业人才实施办法，实施“柔性引才”机制，精准引进产业领军人才和创新创业团队。

做优存量经济。开展第四次全国经济普查，摸查掌握发展现状。深入落实“一企一策”“小升规”等106项精准扶企惠企措施，全力推动传统产业提质升级、做大做强，完成“个转企”1000家。落实“四上企业解三难”等措施，继续开展“四梁八柱”企业认定工作，着力培育一批排头兵企业。支持工业地产发展，推进产业集聚区通用厂房建设，引导中小微企业规范化、园区化、集聚化发展。狠抓品牌建设，继续举办陶瓷、婚纱礼服等专业性、国际性交流展示活动，申报“中国食品名城”，宣传推介“中国瓷都”“中国工艺美术之都”等金字招牌，不断提升潮州品牌的影响力和含金量。推进质量强市建设，支持企业参与各类标准制修订，创建提升国家级出口产品质量安全示范区、枫溪区“日用陶瓷产业全国知名品牌示范区”、古巷镇“卫生陶瓷产业全国知名品牌示范区”，加快市特种设备检测基地建设。

做大增量经济。发挥山海资源和空铁港优势，着力打造沿海沿江沿高铁经济带，推进韩东新城、高铁新城、闽粤经济合作区、凤泉湖高新区等重大平台建设，引领带动樟溪低碳工业园、官塘工业园、潮安特色产业园等产业集聚区加快发展。进一步理顺园区管理体制机制，加强土地征收和配套设施建设，全力打造经济发展新引擎。加大产业共建力度，实施产业链招商、专业招商、潮人招潮商等“精准招商”机制，积极承接粤港澳大湾区等地的产业梯度转移。严把项目“体检准入”关，着重发展新材料、新能源、生物医药、装配式建筑等新兴产业。落实“绿色通道”“保姆式”服务机制，破解“用地难”“审批难”“融资难”问题。按照省关于把东西两翼沿海地区打造成新增长极的战略部署，依托闽粤经济合作区和潮州港，培育发展港口经济、海洋经济，下大力气推动一批百亿级重大产业项目落地投产，努力打造千亿级沿海产业集群。扶持推动已入园落地企业投产量产、增资扩产，加快培育形成新动能。开展总部企业认定工作，引进区域性职能型企业总部，推进总部基地建设，大力培育总部经济业态。

做活做实第三产业。着力在中高端消费、创新引领、绿色低碳、共享经济等领域培育新增长点。大力发展商贸、科技、物流等生产性服务业，加快建设凤凰广场、财富中心等高端商贸综合体，抓好中国工艺美术之都博览园项目。鼓励发展共享经济，稳妥推进共享单车落地运营，加快出租车行业改革，规范网约车市场。开展“信用消费进万家”等活动，培育新兴领域消费市场。支持电子商务发展，建设4个省级电商体验馆，完成电子商务进农村7个镇级、217个村级服务站点建设。大力推进“文化+”，鼓励发展文创产业，力促木雕、潮绣等传统工艺走精品化与规模化融合发展路子。围绕打造潮文化精品城市，加快全域旅游示范区创建工作，推进古城文化旅游“10+4”项目，抓好宋窑大遗址公园、古城博物馆群等项目建设，打造中国瓷都陈列馆、文化科技产业园、中山路工艺一条街、龙湖古寨“一馆一园一街一寨”，推进许驸马府片区和旧府衙片区综合整治、百家修百祠等项目，抓好古城区部分街区成片改造，全面拓展古城旅游的纵深度。持续完善旅游基础设施，提高旅游服务供给能力。统筹整合全市旅游资源，市县区联动发展生态旅游、乡村旅游、红色旅游、工业旅游、节庆旅游、瓷都旅游，举办“潮州美食节”等活动，不断丰富旅游产品。巩固百家旅行社签约成果，加强与国内外各大旅游机构和媒体的深度合作，精准推介古城旅游，不断提高引客入潮成效。

二、聚焦基础设施，完善城市配套

着力实施年度总投资194.3亿元的124个市级重点项目，扩大投资规模，补齐发展短板，完善配套设施，促进城市功能品位提升。

大力推进城镇化建设。加快新型城镇化综合试点工作，探索推动“文商旅”融合发展，抓好意溪、钱东试点小镇建设，推进国家、省、市特色小镇创建工作。按照“多规合一”的总体思路完善城市规划体系，编制实施地下管廊、海绵城市、枫溪区控规等专项规划，全面完成村庄规划编制任务。建立完善土地征收储备工作机制，抓好韩东新城、意溪片区及产业园区的征地工作，完成土地征收3.6万亩，保障土地有效供给。加快中心城区扩容提质步伐，坚持“新老并重”原则，抓好韩东新城开发建设，推进“三旧”改造项目。推进金塘路、东山路一横、恒大城片区配套管网工程。加快构筑管道燃气供应“一张网”，开工建设高压天然气管道等项目。实施1250个电网建设和升级改造项目，突出抓好斗文、归湖、坪溪3个220千伏输变电工程和110千伏凤山输变电工程建设，切实满足城市发展的电力需求。以新一轮农网改造为契机，加快小水电自供区域供电改革。建成充电桩120条。

大力推进交通建设。按照“完善骨干网、织密支线网、提升主干道”的思路，着力打造“江海陆空、互联互通”的立体综合交通体系，构建“粤闽”交通走廊和“汕潮揭梅”区域交通枢纽。实施年度总投资68.5亿元的36个交通重点项目，突出抓好“三站二线一所一广场”7个铁路项目，加快推进潮汕站站改和扩容工程、潮安火车站改造、梅汕客运专线潮州段、潮州动车运用所、饶平站站前广场建设，争取沿海高铁在凤泉湖高新区规划潮州东站，推动广梅汕铁路增建二线及厦深联络线建成通车。开工建设甬莞与沈海高速潮州东联络线，推进大潮高速、潮汕环线高速潮州段无障碍施工。加快潮州东大道、桥东交通综合枢纽等22个道路项目建设，开工建设凤凰大桥，启动东凤大桥立项，确保外环西路、如意大桥延长线、护堤路枫溪段完工通车。加速推进潮州港扩建货运码头、公用航道一期工程等项目，抓好金狮湾公共防波堤工程前期工作。争取省配套资金支持，加大市、县（区）、镇三级财政资金投入，建设“四好农村路”，新改建通村公路120公里。深入推进公交客运行业改革，完善公交客运“一张网”。

大力推进水利建设。以提升水生态环境为目标，全面加快水利基础设施建设，完成“一江两岸四堤”综合整治工程,加快推动引韩济饶供水工程、饶平海堤围达标加固二期工程、粤东灌区续建配套与节水改造工程和省级水利示范县项目建设，力争古枫涝区、澄饶联围、北溪等综合整治和金山大桥下湿地公园提升等项目早日开工。推进农田水利基本建设，加快灌区改造、中小河流治理。深入实施“给排水网”三年建设行动计划，推进村村通自来水工程，抓好北站二路、意东三路排水工程等项目。

大力推进信息化建设。以建设“数字政府”为契机，加快实施“互联网+”和大数据战略，全面提升信息化水平。推进智慧城市工程落地，投放一批智能无人值守便利店，完善智能交通指挥中心，推进“新一代移动警务平台”升级，搭建数字化城市管理平台，推动教育、就业、社保、交通等领域的大数据开发建设，促进各类信息应用便民化。抓好电子政务云平台、政务信息资源共享平台等建设，推进政务服务线上线下融合发展，将网上办事大厅功能延伸到镇（街）村（居）。积极对接浪潮、华为、京东和腾讯等公司，促进大数据、云计算等战略性新兴产业发展。加快发展工业互联网，支持企业“上云用云”。

三、聚焦改革攻坚，提升开放水平

推进更深层次的改革和更高水平的开放，统筹整合优势资源，广泛凝聚发展正能量，实现更好更快发展。

深化供给侧结构性改革。坚决打好防范重大风险攻坚战，深入抓好去产能、去库存、去杠杆、降成本、补短板工作，推动经济发展质量变革、效率变革、动力变革。坚定不移推进国有企业改革，完善出资人权力清单和责任清单，确保国有资产安全运营和保值增值。深化“僵尸企业”出清工作，全面淘汰落后产能。加快启动万山红农场和湘桥东湖片区等棚户区项目建设，完成农村危房改造任务。探索建立多主体供给、多渠道保障、租购并举的住房制度，落实房地产市场调控政策，促进房地产业健康发展。深化投融资体制改革，加强与政策性银行的融资合作，推动韩江投资公司发债筹资，发挥“潮民投”等平台作用，切实扩大有效投资。积极推广PPP模式，引导民间资本参与基础设施建设。提升金融服务实体经济能力，鼓励企业利用资本市场直接融资。推进中小微企业转贷服务工作，降低企业融资成本。推广普惠金融“村村通”经验，优化基层金融服务。完善金融风险预警防控机制，强化风险属地处置责任，严厉打击非法集资，坚决防控互联网金融、涉众金融等风险。加快推进农信社改制，完成组建农商行工作。严防政府债务风险，建立政府债务及财政中长期支出责任登记台账，严格落实地方政府债务限额管理要求，坚决遏制隐形债务增量。强化降成本保障和支撑，全力推进“补短板”项目投资建设。

推动形成全面开放新格局。挖掘原中央苏区县优势资源，加强与邻近苏区县的交流合作。以临港空铁经济合作区设立为契机，推动高铁新城开发建设，深化与汕头、揭阳的对接合作，促进汕潮揭同城化发展。深入推动与中山市的对口合作，推进创新平台建设、“8+1”合作，促进优势产业共融发展。深化与泰国曼谷等友城的交流，争取与欧洲“瓷都”城市缔结为友城。开展“潮文化海外行”系列活动，打造海丝文化重镇。深挖“一带一路”沿线市场潜力，拓宽潮港澳交流合作领域，组织企业参加国内外各类展销会，做大外贸进出口。支持广东工贸通等企业发展外贸综合服务，鼓励跨境电商发展，优化提升外贸业态。

四、聚焦综合治理，优化发展环境

树立环境就是生产力的理念，系统深入开展综合治理，努力营造良好发展环境，优化提升发展软实力。

优化营商环境。完善领导干部挂钩联系服务企业制度，实现规上企业挂钩全覆盖，努力构建亲清新型政商关系。大力营造国际化法治化便利化营商环境，全面实施“1+N”产业扶持政策，着力降低制度性成本、税费成本。深化商事制度改革，落实“多证合一”“证照分离”改革措施。推进国地税征管体制改革，落实国务院六项减免税政策，努力打造便捷式办税服务体系。优化不动产统一登记服务，提升施工图审查效率，取消一批涉企涉民证明事项，健全容缺受理、并联审批、代办专班等服务制度，力促行政审批再提速20%以上。完善工业企业基本信息库，抓好市场主体规范化管理。开展经济户口清理，深入治理“假冒伪劣偷漏骗”行为，营造公平规范竞争环境。

优化城乡环境。坚持“治建管”并重，持续深入推进“六城同创”“治六乱”专项行动，完成环卫体制改革，理顺县区城管执法体制，抓好网格化管理、常态化督查，努力营造整洁有序的城乡环境。抓好肉菜市场、智能停车场、道路“黑底化”等市政工程，完成外环北路绿化提升、一江两岸景观亮化等项目建设，结合人防工程推进社会公共停车场建设，力促城市设施大优化、功能大提升。坚持以潮文化为主题，规划建设一批城市雕塑、立面景观、城市广告和公共设施，切实增强城市的“潮味”和“瓷味”。坚持以人性化和精细化为导向，深入推进厕所革命、街区绿化、交通标识建设，不断提升城市的品位度。编制“十三五”土地整治规划，开展土地“三未”“两违”清理整治，提升节约集约用地水平。

优化平安环境。深化平安潮州建设，加快推进“雪亮工程”，完成“平安潮州”三期视频系统建设，强化城市交通管理，构建立体化、信息化、智能化社会治安和交通管理网络。依法严厉打击和惩治黄赌毒黑拐骗等违法犯罪行为，深化“反邪教”“扫黄打非”进基层工作，大力推动民生警务便民高效。积极推动社会治理重心向基层下移，推进“中心+网格化+信息化”建设，打造湘桥凤新综治中心社会治安共建共治共享新模式，全面推进基层治理“职能综合化、服务网格化、平台智能化、治理社会化”。不断完善预防和化解社会矛盾机制，维护社会和谐稳定。抓好“七五”普法，深化一村（社区）一法律顾问工作。严格落实安全生产责任制，强化企业主体责任落实，深入开展安全生产、燃气管道、地质灾害、火灾隐患等风险点危险源排查治理，全面推进“城中村”、“三合一”场所、高层火灾等消防安全整治，完成5个消防站和200个市政消防栓建设，坚决遏制重特大安全事故发生。推进湘桥区创建省级食品安全示范区工作，抓好“放心肉菜示范超市”创建，严厉打击食品药品违法行为，确保群众饮食用药安全。

优化生态环境。坚决打好污染防治攻坚战，完成环保机构监测监察执法垂直体制改革，推进环境保护规划纲要修编，抓好生态保护红线划定工作，开展第二次污染源普查，加强大气、水、土壤和生态监测网络建设。推进扬尘污染综合治理工作，狠抓陶瓷垃圾、建筑垃圾收集处理点建设，推动《潮州市扬尘污染防治条例》立法，制定实施露天焚烧监管等大气治理专项制度，全力打赢蓝天保卫战。全面推行河长制、湖长制，强化对韩江、黄冈河等各类水体的管理保护，推进饮用水源保护区调整。铁腕推进中央环保督察反馈意见整改落实，抓好枫江流域综合治理，加快污水处理设施及配套管网建设，完成市一污扩容改造工程，抓好镇级垃圾填埋场治理，推进市区环保发电厂、饶平宝斗石生活垃圾填埋场建设。落实国家海洋督察反馈意见整改，抓好西澳岛生态保护与修复项目建设。加快“国家森林城市”创建步伐，推进桉树林改造，实施新一轮绿化潮州大行动，完成森林碳汇造林1.1万亩，新增绿化美化乡村73个。

五、聚焦共建共享，增进民生福祉

坚持在发展中保障和改善民生，大力发展各项社会事业，不断满足人民日益增长的美好生活需要。

保障和改善民生。实施就业优先战略，优化公共就业服务，开展劳动力技能培训。推进创业孵化基地建设，加大创业担保贷款扶持力度，提高创业带动就业实效，新增城镇就业1.5万人。推进社保扩面征缴，建设多层次社会保障体系。统筹提高企业职工、城乡居民养老保险待遇水平，完善大病保险制度，强化健康保障能力。健全社会救助、社会福利、慈善救济机制，提高城乡低保、特困人员供养标准和补贴水平，保障困难群众基本生活。坚决打好精准脱贫攻坚战，全面落实扶贫保障政策，加快推进产业、就业、金融、教育、医疗等扶贫措施，千方百计提高贫困户收入水平，实现“两不愁、三保障”目标，确保全部相对贫困村出列、全部相对贫困人口脱贫。深化45个省定贫困村“三清理、三拆除、三整治”工作，加快村道硬化、集中供水、雨污分流、垃圾处理、电网改造等基础设施建设，全面达到省整治标准。

积极实施乡村振兴战略。坚持农业农村优先发展，按照产业兴旺、生态宜居、乡风文明、治理有效、生活富裕的总要求推动农村振兴发展。以基层党建为引领，提升农村治理水平；以村庄规划为引领，提升农村建设水平；以产业项目为引领，提升农村发展水平。全面完成省级新农村连片示范建设任务并通过省绩效评估。深入推进农业供给侧结构性改革，完成农村土地确权工作任务，抓好潮安区农村集体产权制度改革试点工作。坚持质量兴农、绿色兴农，完成垦造水田、高标准基本农田建设和粮食生产功能区划定任务，完善地方储备粮管理体系，确保粮食安全。发展特色农业，大力提升1.1万亩标准化生态茶园、浮滨狮头鹅良种繁育示范基地、潮安万亩淡水养殖示范区，推进茶叶、铁皮石斛、果蔬“三带一点”建设，力促特色农业向优势主产区聚集。建设农业产业链数据服务平台，大力培育农业合作社，促进农村一二三产业融合发展。充分运用第三次全国农业普查和自然村落人文普查成果，服务乡村振兴发展。

提高基本公共服务水平。以创建教育现代化先进市为抓手，推进学校布局调整优化，扶持和规范社会力量办学，扩大优质教育资源供给。加快市绵德小学、市绵德幼儿园等一批公办学校易地重建。加强与名校合作办学，推进光正实验学校、暨大潮州附属实验学校、华师潮州附属学校建设。加快潮州卫生健康职业学院创办和潮汕高等研究院筹建。完善市县镇村四级公共文化设施网络，推动“博物馆之城”创建，规划建设潮剧艺术中心、体育中心、体育公园，加快“三馆一站”建设，实现基层综合性文化服务中心全覆盖。加大潮州传统文化传承保护力度，依托中国刺绣艺术研究院等平台，培育壮大非遗传承人队伍，联合举办非物质文化遗产可持续发展国际会议等学术研讨交流活动，加强非遗保护传承。推进“国家卫生城市”创建工作，深化医药卫生体制改革，提高基本公共卫生服务均等化水平。加快市中心医院新院项目、市妇幼保健院改扩建项目建设，落实市人民医院后续扩建用地，争取择点易地新建市中医医院，加快潮安区妇幼保健院、饶平县人民医院等6所县级医院改扩建工程和村卫生站公建规范化建设。提高优生优育服务水平和妇幼保健能力，促进人口均衡发展。加快发展养老服务业，确保养老床位数达到每千名老人35张。结合省的部署安排和人民群众的期望诉求，加大财政民生投入力度，重点落实好“医、食、住、行、学、养”等民生保障，全力办好十件民生实事，努力让人民群众的获得感、幸福感、安全感更加充实、更有保障、更可持续。

与此同时，加强双拥优抚安置工作，创新军民融合深度发展机制，大力支持国防和军队建设。以“聚心聚力、共创共享”为目标，进一步团结海内外潮州人共同发展。加快推进更高水平的气象现代化建设。支持工会、共青团、妇联等人民团体工作，进一步做好民族宗教、广播电视、新闻出版、对台、人防、防震、统计、物价、档案、地方志、残疾人、红十字等工作。

为统筹推进经济社会各项事业加快发展，更好保障和改善民生，继续坚持重点工作项目化、重点项目责任化，参照“六个十”项目模式，全力推动实施10个以上重大交通项目、10个以上重大产业项目、10个以上重大水利项目、10个以上重点环保项目、10个以上学校项目、10个以上医院项目、10个以上重点旅游项目、10个以上城区改造提升项目等“八个十”项目。坚持条块结合、统筹联动，按照项目目标化、目标节点化、节点责任化的要求，调动各方力量，集聚优势资源，全力以赴打好一场重点项目攻坚战。

六、聚焦党的建设，提高政府效能

认真贯彻党的十九大精神，深入落实习近平总书记对广东工作的重要指示批示精神，以严实作风开展“大学习、深调研、真落实”工作，切实推动习近平新时代中国特色社会主义思想落地生根、开花结果。坚持全面从严治党，以“全面增强执政本领”为要求导向，系统深入推进党的建设，扎实开展“两学一做”“不忘初心、牢记使命”主题教育，进一步健全机制、完善制度、正风肃纪、提高效率，着力建设干净担当、务实高效的服务型政府。

强化法治思维，切实做到依法行政。深入推进法治政府建设，加强政府立法和规范性文件制订管理工作，完善科学依法决策机制。强化执法监督，营造公平文明执法环境。健全政务公开考核制度，广泛听取社会各方面意见和建议。认真办理人大代表建议和政协委员提案，自觉接受人大、政协监督，构建决策科学、执行坚决、监督有力的权力运行机制。

强化服务意识，切实做到务实高效。转变政府职能，深化简政放权，创新监管方式，着力压减市级权责清单，增强政府服务力和公信力。推进行政机构改革，提高编制使用效率。深化“一门式、一窗式、一网式”政务服务整合共享，简化程序，提高效率。推进基层公共服务平台建设，打通公共服务“最后一公里”。

强化使命担当，切实做到奋发有为。以提高“八大执政本领”为目标要求，广泛组织开展政策法规、专业技能等学习培训，不断提高各级干部的综合素质和业务能力。建立健全重点工作责任落实机制，采取“三定一督办”“钉钉子”等工作法，结合责任表、“鱼刺图”等抓手，定量定时推动各项工作。进一步健全大督查工作机制，通过跟踪督查、动态管理，力促各项工作落地落实。实施政府绩效考核奖励机制，完善容错纠错机制，旗帜鲜明为有力有为的单位和干部撑腰鼓劲。强化监督问责，坚决纠正不作为、乱作为等问题。

强化廉政建设，切实做到风清气正。严明政治纪律和政治规矩，坚决维护习近平总书记在党中央和全党的核心地位，坚决维护以习近平同志为核心的党中央权威和集中统一领导，始终在思想上政治上行动上同以习近平同志为核心的党中央保持高度一致。严格落实廉政建设主体责任，严肃查处腐败案件，全力打造廉洁政府。贯彻落实中央八项规定实施细则精神，坚持不懈改进工作作风，切实营造风清气正的干事创业氛围。坚持厉行节约，缩减“三公”经费，严控一般性支出，切实降低行政运行成本。加强对重点领域、重点项目、重点资金的审计与监察，确保权力在阳光下运行。

各位代表，新时代催人奋进，新征程使命光荣。让我们紧密团结在以习近平同志为核心的党中央周围，高举习近平新时代中国特色社会主义思想伟大旗帜，按照习近平总书记对广东工作的重要指示批示精神，在省委、省政府和市委的坚强领导下，不忘初心，牢记使命，以雷厉风行的干劲、久久为功的韧劲、敢为人先的拼劲，开拓进取，奋勇拼搏，为开创新时代潮州振兴发展新局而努力奋斗！
